# Insights into the bacterial community composition of farmed *Caulerpa lentillifera*: A comparison between contrasting health states

**DOI:** 10.1002/mbo3.1253

**Published:** 2021-11-25

**Authors:** Germán A. Kopprio, Nguyen D. Luyen, Le Huu Cuong, Tran Mai Duc, Anna Fricke, Andreas Kunzmann, Le Mai Huong, Astrid Gärdes

**Affiliations:** ^1^ Department of Ecohydrology and Biogeochemistry Leibniz Institute of Freshwater Ecology and Inland Fisheries Berlin Germany; ^2^ Institute of Natural Product Chemistry Vietnam Academy of Science and Technology Hanoi Vietnam; ^3^ Vietnam Academy of Science and Technology Graduate University of Science and Technology Hanoi Vietnam; ^4^ Nha Trang Institute of Technology Research and Application Vietnam Academy of Science and Technology Nha Trang Vietnam; ^5^ Department of Plant Quality and Food Security Leibniz Institute of Vegetable and Ornamental Crops Großbeeren Germany; ^6^ Department of Ecology Leibniz Centre for Tropical Marine Research Bremen Germany; ^7^ University of Applied Sciences Bremerhaven Germany; ^8^ Department of Biosciences, Alfred Wegener Institute Helmholtz Centre for Polar and Marine Research Bremerhaven Germany

**Keywords:** 16S rRNA gene, aquaculture, green caviar, sea grape, seaweed, Vietnam

## Abstract

The bacterial communities of *Caulerpa lentillifera* were studied during an outbreak of an unknown disease in a sea grape farm from Vietnam. Clear differences between healthy and diseased cases were observed at the order, genus, and Operational Taxonomic Unit (OTU) level. A richer diversity was detected in the diseased thalli of *C. lentillifera*, as well as the dominance of the orders Flavobacteriales (phylum Bacteroidetes) and Phycisphaerales (Planctomycetes). *Aquibacter, Winogradskyella*, and other OTUs of the family Flavobacteriaceae were hypothesized as detrimental bacteria, this family comprises some well‐known seaweed pathogens. *Phycisphaera* together with other Planctomycetes and *Woeseia* were probably saprophytes of *C. lentillifera*. The Rhodobacteraceae and *Rhodovulum* dominated the bacterial community composition of healthy *C. lentillifera*. The likely beneficial role of *Bradyrhizobium, Paracoccus*, and *Brevundimonas* strains on nutrient cycling and phytohormone production was discussed. The bleaching of diseased *C. lentillifera* might not only be associated with pathogens but also with an oxidative response. This study offers pioneering insights on the co‐occurrence of *C. lentillifera*‐attached bacteria, potential detrimental or beneficial microbes, and a baseline for understanding the *C. lentillifera* holobiont. Further applied and basic research is urgently needed on *C. lentillifera* microbiome, shotgun metagenomic, metatranscriptomic, and metabolomic studies as well as bioactivity assays are recommended.

## Graphical abstract

The co‐occurrence of bacteria in sea grape (green caviar) *Caulerpa lentillifera* under two health state conditions was studied. The role of potentially detrimental and beneficial bacteria was discussed. A baseline for understanding the *C. lentillifera* holobiont was presented.

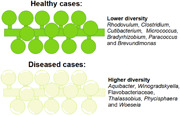

## 1 INTRODUCTION

The sea grape or green caviar *Caulerpa lentillifera* J. Agardh 1837 (Ulvophyceae, Bryopsidales) is a green macroalga widely cultivated in tropical countries of the Indo‐Pacific region, and its market as a seafood delicacy has been expanded drastically in the last decades. Not only *C. lentillifera* unique texture, flavor, and appearance which let it resemble caviar (e.g., Ly et al., 2021), but also its elevated contents of polyunsaturated fatty acids, antioxidants, vitamins, and trace elements, comparatively higher than other edible macroalgae (de Gaillande et al., 2017; Paul et al., 2014; Saito et al., 2010; Yap et al., 2019), makes it a valuable functional food with exponentially increasing demand (Terada et al., 2021). Moreover, the combination of the high nutritional quality with the simple and low‐cost cultivation transform *C. lentillifera* into a promising candidate to enhance food security in tropical countries (de Gaillande et al., 2017; Stuthmann et al., 2021). However, one of the major factors contributing to the worldwide decline of macroalgal populations are microbial diseases (Egan et al., 2014) and *C. lentillifera* aquaculture is currently endangered by different outbreaks (Liang et al., 2019).

Macroalgae provide a nutrient‐rich niche for microbes and some bacteria are capable to invade algal tissue and causing disease by degrading complex polymers. Agarases, carrageenases, alginases, fucoidanases, fucanases, mannanases, cellulases, and pectinases are depolymerizing enzymes detected in several marine bacteria, responsible for the breakdown of algal cell walls (Goecke et al., 2010). Moreover, the low genetic diversity of cultivated algae and their tight distribution in aquaculture settings facilitate the rapid spread of detrimental microbes (Valero et al., 2017; Ward et al., 2020). Several bacterial diseases have been reported in commercial macroalgae such as the rote spot and hole‐rotten diseases caused by *Pseudomonas* spp. in *Saccharina japonica*, rotten thallus syndrome by *Vibrio* sp. in *Gracilaria verrucosa*, or Anaaki by *Flavobacterium* spp. in *Pyropia yezoensis* (reviewed by Egan et al., 2014; Goecke et al., 2010; Ward et al., 2020). Nevertheless, the role of microbes is not only detrimental but can also be beneficial.

Some bacteria are essential for algal health, growth, and development. Beneficial bacteria attached to macroalgae induce morphogenesis, fix nitrogen, produce phytohormones, release antifouling and antimicrobial compounds, transport metabolites and nutrients, detoxify pollutants, contribute to algal reproduction, and are key for their adaptation to new environments (Aires et al., 2013; Hollants et al., 2013; R. P. Singh & Reddy, 2014). The relationship between macroalgae and bacteria is generally mutualistic, and bacteria are mainly benefited from organic substrates for their metabolism and a stable microenvironment protected from predators and changing environmental conditions. The relationship between macroalgae and bacterial communities is so tight and reciprocal that the whole entity is considered a holobiont (Arnaud‐Haond et al., 2017; Califano et al., 2020; Egan et al., 2013). Furthermore, the bioactive compounds produced by microbes in the holobiont have an enormous potential for drug discovery and biotechnological applications (Friedrich, 2012; Luyen et al., 2019).

Despite the commercial importance of *C. lentillifera* and its relevance for human nutrition under future climate‐driven and overpopulation scenarios, studies about their microbiome are very limited. Liang et al. (2019) described a disease in *C. lentillifera* in Chinese aquaculture systems characterized by a dark‐green biofouling, pink‐colored and missing ramuli. In this particular case, Bacteroidetes and Cyanobacteria dominated the bacterial communities of diseased *C. lentillifera*. Although recent advances in the study of the macroalgal microbiome using next‐generation sequencing (NGS) techniques, the information is still limited and in several cases derived from culture methods with their intrinsic limitations (Friedrich, 2012). The 16S ribosomal RNA (rRNA) gene approach offers broader insights into the natural composition of bacterial communities attached to algae and plays an important role in understanding the holobiont during disease (Egan et al., 2014; Kopprio et al., 2021). The aims of this study were: (1) to characterize the bacterial communities of *C. lentillifera* in a Vietnamese sea grape farm and (2) to assess the bacterial community composition under two different health conditions. We hypothesize a higher diversity and a dominance of Bacteroidetes in the bacterial communities of diseased *C. lentillifera*.

## 2 METHODS

### 2.1 Culture conditions, sampling, and disease symptoms

**Figure 1:**
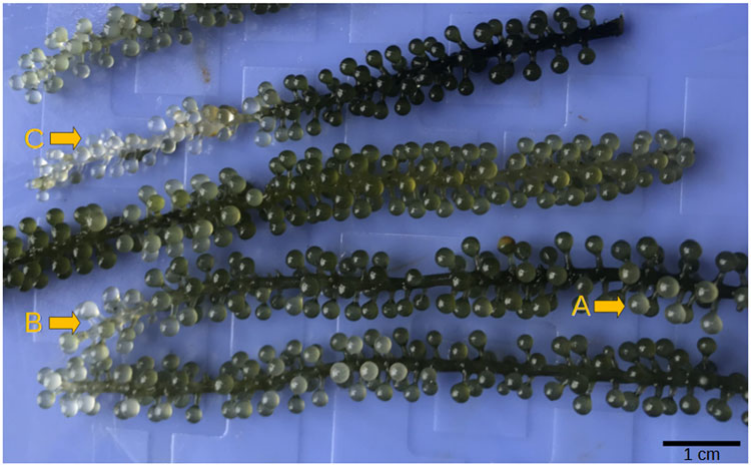
Diseased *Caulerpa lentillifera* from the Vietnamese aquaculture. (A) White biofilm on the external circumference of the ramuli. (B) Bleaching of the ramuli. (C) Bleaching, shrinking, and loss of ramuli

*C. lentillifera* thalli were sampled in the facilities of the company VIJA at Van Phong Bay (N 12° 35′ 17.67″, E 109°13′ 39.76″) in the central‐eastern coastline of Vietnam. Water temperature in the ponds reached ∼31°C in May, with pH values of ∼8.1, conductivity ∼53 mS cm^−1^, and dissolved oxygen ∼5.6 mg L^−1^. The sea grapes were cultivated in ponds, which were used previously for shrimp farming and then placed for cleaning in tanks a few days before processing. The ponds and tanks were covered with shade cloths to avoid direct sunlight. Six healthy and seven diseased thalli of *C. lentillifera* from different tanks, originally harvested from the same pond, were collected during an outbreak of an unknown disease. In contrast to the bright green color of healthy individuals, the diseased *C. lentillifera* showed a white biofilm on the external circumference of the vesicular ramuli (Figure 1, Arrow A) followed by a marked tissue discoloration or bleaching (Figure 1, Arrow B). After the described symptoms, the diseased algae lost their turgid appearance and ramuli, and thereafter decayed (Figure 1, Arrow C).

### 2.2 DNA extraction and amplification

*C. lentillifera* thalli were collected in sterile vials and preserved at −20°C until further analysis. Within a few days, the samples were transported frozen and DNA was extracted according to Griffiths et al. (2000) under the laboratory conditions of the Leibniz Centre for Tropical Marine Research. For every sample, ∼10 ramuli together with the supporting rachis were immersed in 600 µl of hexadecyltrimethylammonium bromide with 60 µl of 10% sodium dodecyl sulfate and 60 µl of 10% *N*‐lauroylsarcosine. Glass beads were added and the tissue was homogenized in a FastPrep at 5 m s^−1^. Homogenates were rinsed with 600 µl of phenol–chloroform–isoamyl alcohol (25:24:1) and centrifuged at 16,000*g* and 4°C for 10 min. The upper aqueous layer was transferred to a clean reaction tube, mixed with two volumes of 30% polyethylene glycol 6000 and 1.6 M NaCl, and incubated at 4°C for 120 min. Subsequently, the sample was centrifuged at 17,000*g* and 4°C for 90 min, the supernatant was carefully removed and the pellet was washed with ice‐cold 70% ethanol. The pellet was air‐dry at 37°C for a few minutes, dissolved in 20 μl polymerase chain reaction grade water, and stored at −20°C. The hypervariable region V3‐V4 of the 16S rRNA gene was amplified with the set of primers according to Klindworth et al. (2013): Bact‐341F (5ʹ−3ʹ: CCT ACG GGN GGC WGC AG) and Bact‐785R (GAC TAC HVG GGT ATC TAA KCC)

### 2.3 Sequencing, bioinformatics, and statistical analysis

The amplicon V3‐V4 of the 16S rRNA gene was sequenced with a 2 × 300‐bp paired‐end run on an Illumina MiSeq platform. Sequencing, removal of primer sequences, and demultiplexing were conducted by the company LGC genomics. Sequences were trimmed and merged using Trimmomatic v0.36 (Bolger et al., 2014) and PEAR v0.9.8 (J. Zhang et al., 2014), respectively. A total of ∼373,000 reads with a mean of 28,700 reads per sample were detected after primer removal, and a total of ~272,000 reads with a mean of 20,900 reads remained after merging. Operational Taxonomic Units (OTUs) were clustered by minimum entropy decomposition (MED) MED v2.1 (Eren et al., 2015) and their representatives were submitted to SilvaNGS (v132; https://ngs.arb-silva.de/silvangs/) using a sequence similarity of one for clustering and the remaining variables as default. Singleton, doubleton, and sequences from mitochondria, chloroplasts, and archaea were removed from the analysis. Demultiplexed and primer‐clipped sequences were deposited at the European Nucleotide Archive using the data brokerage service of the German Federation for Biological Data (Diepenbroek et al., 2014) with the number PRJEB42826, in compliance with the minimal information about any (x) sequence standard (Yilmaz et al., 2011).

Pooling of taxa, removal of OTUs with poor alignment quality, and relative sequence abundance and diversity calculations were conducted in R v4.0.5 (R Core Team, 2021) and additional packages such as vegan (Oksanen et al., 2019) and iNEXT (Hsieh et al., 2019). The rarefaction curves were performed with iNEXT, reached a plateau and the mean number of reads per sample used for statistical analyses was ∼17,000. The diversity indexes: Chao, Shannon, and Inverse Simpson were calculated with iNEXT. Relative sequence abundances of the main orders, genera, and diversity indexes between the diseased and healthy cases were compared with a Mann–Whitney test. Data were transformed (*x* = log [(OTU number + OTU mean_sample_)/OTU mean_sample_]), a Bray Curtis similarity matrix was calculated, and differences between the states at order and OTU level were evaluated using permutational multivariate analysis of variance (PERMANOVA). In case of significant differences, similarity percentage analysis (SIMPER) was performed to detect the main taxa contributing to dissimilarities. Samples at the OTU level were ordinated by nonmetric multidimensional scaling (NMDS). A heat map based on the 60 most abundant OTUs, covering 64 ± 11% of the total relative sequence abundance, was performed with the software XLSTAT‐Ecology (Addinsoft, 2018) using the default parameters. The dendrogram at the top of the heat map indicated similarity between samples, while the dendrogram on the left side showed similarity between OTUs. Graphics and statistics were performed with R 4.0.5, Xact 7.21, PRIMER v6 + PERMANOVA, and XLSTAT‐Ecology.

## 3 RESULTS

**Figure 2:**
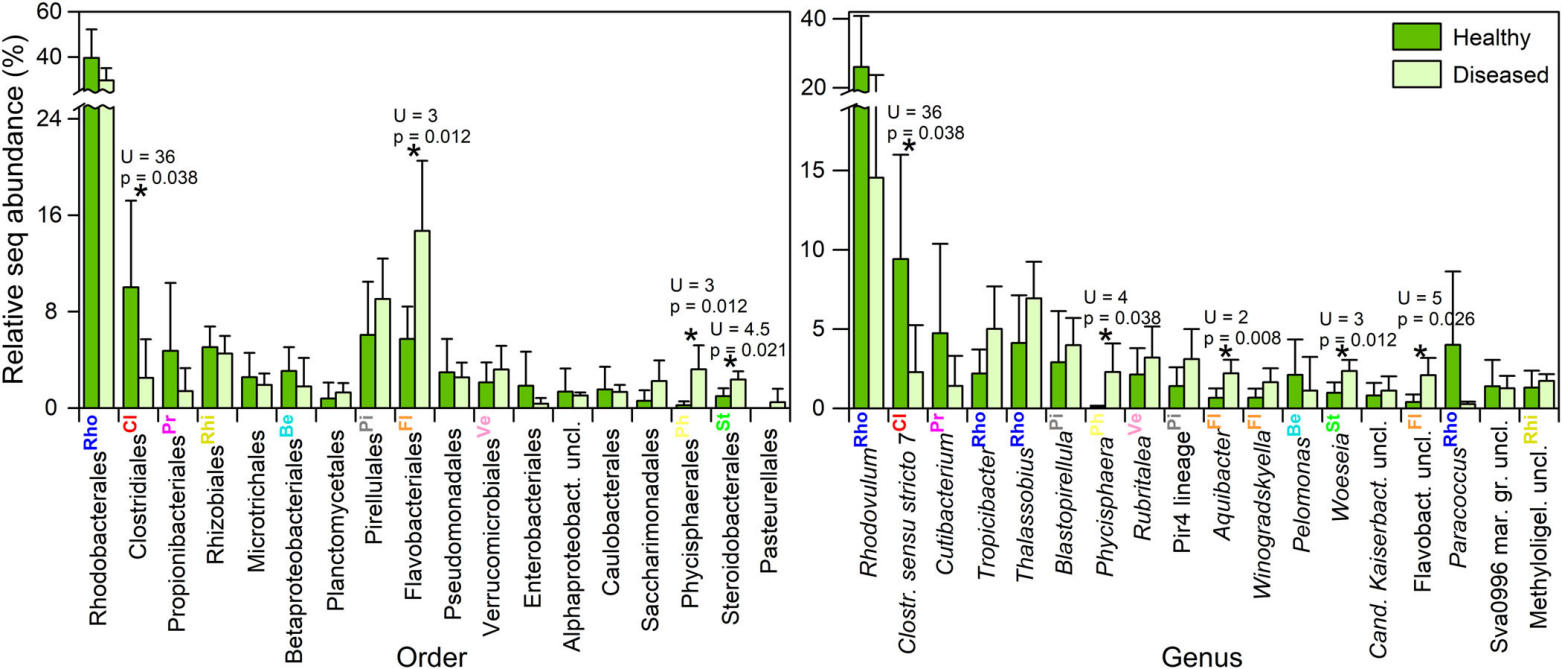
Mean relative sequence abundance (±standard deviation) of the main bacterial orders and genera in healthy and diseased individuals of the sea grape *Caulerpa lentillifera*. *Significant differences at *p* < 0.05 according to the Mann–Whitney test. Caption superscript and color show correspondence between order and genus (e.g., ^Rho^ = Rhodobacterales). Aphaproteobact., Alphaproteobacteria; *Cand. Kaiserbact*., *Candidatus Kaiserbacteria; Clostr*., *Clostridium*; Flavobact., Flavobacteriaceae; mar. gr., marine group; Methyloligel., Methyloligellaceae; uncl., unclassified

After data curation, the means of the total OTU number per sample were 12,200 for the healthy cases and 21,300 for the diseased cases. A total of 810 unique OTUs were detected in *C. lentillifera*, from which 400 were shared between both cases and 380 were exclusively found in diseased *C. lentillifera*. The order Rhodobacterales (class Alphaproteobacteria) and its genus *Rhodovulum* presented the highest relative sequence abundance in *C. lentillifera* at each respective taxonomic level (Figure 2). The Rhodobacteraceae was the only family detected within the order Rhodobacterales. The mean relative abundance of Clostridiales (phylum Firmicutes) was approximately three times significantly higher in healthy *C. lentillifera* as confirmed by the Mann–Whitney test (Figure 2). The mean abundance of Flavobacteriales of the phylum Bacteroidetes was about two times higher in diseased *C. lentillifera* and significant differences were observed. The family Flavobacteriaceae dominated the order Flavobacteriales and comprised 99.7% of the OTUs for this order. The order Phycisphaerales (phylum Planctomycetes) and Steroidobacterales (class Gammaproteobacteria) characterized diseased *C. lentillifera* thalli. The general trend observed at order level was similar at genus level: The relative sequence abundance of *Clostridium sensu stricto* 7 (Clostridiales) was also three times higher in healthy than in diseased cases, while *Aquibacter* and Flavobacteriaceae unclassified (Flavobacteriales), *Phycisphaera* (Phycisphaerales) and *Woeseia* (Steroidobacterales) were typical of diseased cases. Without significant differences, other abundant genera were *Rhodovulum, Cutibacterium*, and *Paracocccus* for healthy *C. lentillifera*, while *Thalassobius, Tropicibacter*, and *Blastopirellula* for diseased *C. lentillifera*.

PERMANOVA revealed significant differences between the healthy and diseased cases at the order level (Pseudo‐*F* = 3.89, *p* = 0.016). According to SIMPER analyses, the total dissimilarity between healthy and diseased cases was 41.3%. The orders which presented significant differences in the Mann–Whitney test were also important contributors to the dissimilarities between healthy and diseased thalli (Table 1). Other relevant orders with higher percentages of dissimilarities but without significant differences were Propionibacteriales and Betaproteobacteriales for the healthy cases, while Saccharimonadales and Verrucomicrobiales were relevant for the diseased cases. Furthermore, PERMANOVA detected significant differences at the OTU level between healthy and diseased *C. lentillifera* (Pseudo‐*F* = 3.12, *p* = 0.015). According to SIMPER analysis, the total dissimilarity at OTU level was 71.3% and the main OTUs contributing to this value are detailed in Table 1.

**Table 1:** Principal orders and OTUs in the sea grape C*aulerpa lentillifera* contributing to the highest dissimilarity values (41% and 71% of the total dissimilarity, respectively) according to SIMPER analyses

	Healthy		Diseased	
	Taxa	%	Taxa	%
Orders	Clostridiales^Cl^	5.3	Phycisphaerales^Ph^	4.4
	Propionibacteriales^Pr^	4.0	Saccharimonadales	3.3
	Betaproteobacteriales^Be^	3.1	Flavobacteriales^Fl^	3.2
	Micrococcales^Mc^	2.6	Verrucomicrobiales^Ve^	2.8
	Enterobacteriales^En^	2.5	Pirellulales^Pi^	2.6
	Microtrichales^Mt^	2.4	Planctomycetales^Pl^	2.6
	Bacillales^Ba^	2.2	Caulobacterales^Ca^	2.3
	Chitinophagales^Ch^	1.7	Steroidobacterales^St^	2.3
OTUs	*Paracoccus* 2980	0.6	*Phycisphaera* 3117^Ph^	0.6
	*Spongiimonas* 0219^Fl^	0.5	Methyloligellaceae uncl. 0867	0.5
	*Clostridium sensu stricto* 7 4975^Cl^	0.5	*Thalassobius* 2935	0.5
	*Cutibacterium* 2507^Pr^	0.5	Pir4 lineage 0189^Pi^	0.5
	*Aliihoeflea* 1742	0.5	Flavobacteriaceae uncl. 2304^Fl^	0.5
	*Pelomonas* 1964^Be^	0.5	*Aquibacter* 3662^Fl^	0.5
	*Brevundimonas* 1698^Ca^	0.5	JGI 069‐P22 0837 uncl.	0.5
	*Bradyrhizobium* 1899	0.4	*Thalassobius* 1635	0.5
	*Proteus* 2600^En^	0.4	*Winogradskyella* 2272^Fl^	0.5
	*Paracoccus* 4667	0.4	*Rubritalea* 2117^Ve^	0.5
	*Clostridium sensu stricto* 7 1787^Cl^	0.4	*Aureicoccus* 3615^Fl^	0.5
	*Micrococcus* 3447^Mc^	0.4	*Woeseia* 0634^St^	0.4
	*Rubritalea* 3432^Ve^	0.4	*Blastopirellula* 1161^Pi^	0.4
	*Woeseia* 3512^St^	0.4	*Coraliomargarita* 3905	0.4
	*Suttonella* 1166	0.4	*Psychrobacter* 1144	0.4
	*Afipia* 1915	0.4	Cyclobacteriaceae uncl. 1240	0.4
	*Ralstonia* 0332^Be^	0.3	*Ulvibacter* 0198^Fl^	0.4
	*Escherichia*‐*Shigella* 2098^En^	0.3	Alphaproteobacteria uncl.1689	0.4
	*Staphylococcus* 0913^Ba^	0.3	Sva0996 1388^Mt^	0.4
	*Kocuria* 3438^Mc^	0.3	*Aquibacter* 2359^Fl^	0.4
	*Enhydrobacter* 0637	0.3	*Blastopirellula* 3839^Pi^	0.4
	Methyloligellaceae uncl. 3194	0.3	Pir4 lineage 0659^Pi^	0.4
	*Vibrionimonas* 3429^Ch^	0.3	*Thalassobius* 4280	0.4
	*Blastopirellula* 3811^Pi^	0.3	*Phycisphaera* 3114^Ph^	0.4

*Note*: Taxa are grouped based on their dominance in healthy or diseased cases. Caption superscript: indicates correspondence between order and genus (e.g., ^Cl^ = Clostridiales). The number after each genus refers to the sequence number or MED node.

Abbreviations: OTU, Operational Taxonomic Unit; uncl., unclassified.

**Figure 3:**
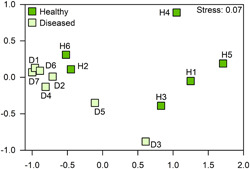
Nonmetric multidimensional scaling ordination of healthy and diseased individuals of the sea grape *Caulerpa lentillifera* at Operational Taxonomic Unit level

The OTUs of order Clostridiales, typical of healthy *C. lentillifera*, were *Clostridium sensu stricto* 7 sq (sequence number or MED node) 4975 and sq 1787 (Table 1). Other OTUs dominating with elevated percentages of dissimilarity in the healthy cases were those of the genera: *Paracoccus, Spongiimonas, Cutibacterium, Aliihoeflea, Pelomonas, Brevundimonas*, and *Bradyrhizobium*. In the case of diseased cases, the major OTUs were those of Flavobacteriaceae unidentified, *Aquibacter, Winogradskyella, Aureicoccus*, and *Ulvibacter* for the order Flavobacteriales (all belonging to the family Flavobacteriaceae), *Phycisphaera* for the order Phycisphaerales and *Woeseia* for the order Steroidobacterales. Other OTUs with elevated percentages of dissimilarity in the diseased cases were Methyloligellaceae unclassified sq 0867, *Thalassobius* sq 2935, Pir4 lineage sq 0189, and JGI 069‐P22 unclassified sq 0837. A list with other relevant OTUs according to their dissimilarity is presented in Table 1. Moreover, the NMDS ordination of *C. lentillifera* samples at the OTU level (Figure 3) suggested contrasting bacterial communities between healthy and diseased cases.

**Figure 4:**
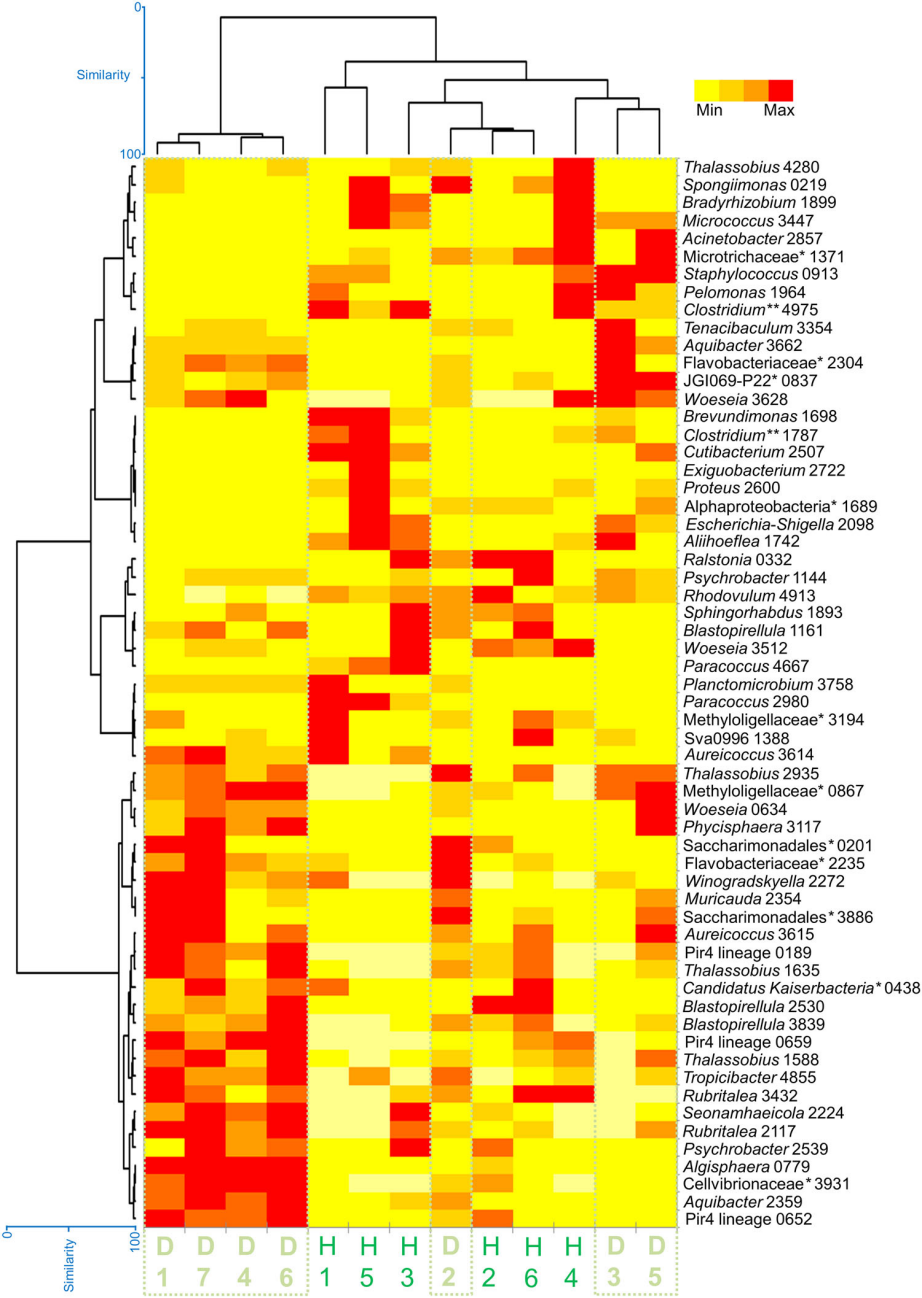
Heatmap of the main Operational Taxonomic Units on healthy (H) and diseased (D) individuals of the sea grape *Caulerpa lentillifera*. (*) Unclassified (**) *sensu stricto 7*. The number after each genus refers to the sequence number or minimum entropy decomposition node

**Figure 5:**
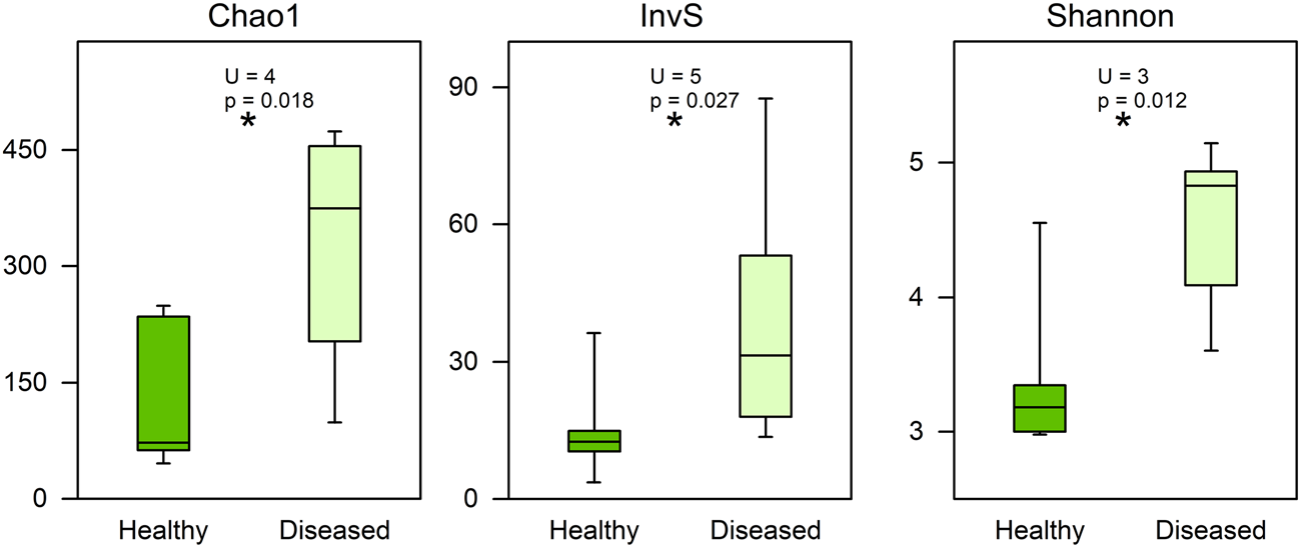
Diversity comparisons between healthy and diseased states of *Caulerpa lentillifera* according to the Chao1, Inverse Simpson (InvS), and Shannon indexes. *Significant differences at *p* < 0.05 according to the Mann–Whitney test

The heat map (Figure 4) showed a similar cluster of four diseased *C. lentillifera*, while a variable ordination of the other cases. OTUs associated exclusively with diseased status were *Aquibacter* sq 3662, Flavobacteriaceae unclassified sq 2304, *Woeseia* sq 0634, *Phycisphaera* sq 3117, and *Muricauda* sq 2354; while those exclusively detected in the healthy status were *Bradyrhizobium* sq 1899, *Paracoccus* sq 4667, and *Paracoccus* sq 2980. OTUs of the genera JGI 069‐P22 unclassified, *Thalassobius*, Saccharimonadales unclassified, Flavobacteriaceae unclassified, *Winogradskyella, Aureicoccus*, Pir4 lineage, *Tropicibacter, Algisphaera*, Cellvibrionaceae unclassified, and *Aquibacter* dominated mainly in the diseased cases. Other OTUs which dominated but not exclusively in the healthy cases are displayed in the heat map. The box plots of the diversity indexes (Figure 5) evidenced higher values in diseased *C. lentillifera* and significant differences according to Mann–Whitney comparisons.

## 4 DISCUSSION

### 4.1 Main composition

The bacterial communities of *C. lentillifera* presented a similar composition as other green algae at higher taxonomic levels. The dominance of Alphaproteobacteria and Bacteroidetes has been reported in green macroalgae and some *Caulerpa* species (Aires et al., 2013; Friedrich, 2012; Singh & Reddy, 2014). The order Rhodobacterales (Alphaproteobacteria) and family Rhodobacteraceae dominated the bacterial communities of *C. lentillifera* in this particular case from the Vietnamese aquaculture. The bacterial communities at lower taxonomic levels are generally highly variable in seaweeds and their composition may be associated with a particular function within the holobiont. The microbiome composition depends generally on the geographic location, environmental factors, seaweed tissues, developmental stage, and even among local individuals (reviewed by Friedrich, 2012). Burke et al. (2011) explain the pattern of bacterial colonization on green macroalgae with the competitive lottery model, according to this model different species with similar functional traits can occupy the same niche depending on a stochastic probability. The highest relative abundance of Rhodobacterales coincided with the results obtained in *C. lentillifera* from a study case in China (Liang et al., 2019); nevertheless, one of the differences was in the dominant genus within Rhodobacterales: *Leisingera* in contrast to *Rhodovulum* in our study.

### 4.2 Potential detrimental bacteria

The pattern of higher diversity in diseased *C. lentillifera* was also observed in a case study from China (Liang et al., 2019), where the spherical ramuli turned pink‐red, detached, and decayed, and the orders Flavobacteriales (Bacteroidetes), Phycisphaerales, and Cellvibrionales dominated the thalli of diseased *C. lentillifera* (Liang et al., 2019). With a different coloration, a similar pattern was observed in our study: Flavobacteriales, Phycisphaerales, and an unidentified genus of the family Cellvibrionaceae were typical of diseased *C. lentillifera*. The family Flavobacteriaceae has the potential to break down algal walls, invade tissues, and cause disease under certain conditions. Bacteroidetes and particularly Flavobacteriaceae are degraders of complex biopolymers like sulfated polysaccharides from algal tissues (Jain et al., 2019; Yilmaz et al., 2016).

Many members of the Flavobacteriaceae have been confirmed or suspected as causative agents of seaweed diseases (Goecke et al., 2010; Kumar et al., 2016; Ward et al., 2020). In our particular case, we hypothesize as potential detrimental bacteria, strains of *Aquibacter, Winogradskyella, Aureicoccus*, Ulvibacter, and *Muricauda*. For example, *Winogradskyella* species have been suggested as detrimental bacteria on *P. yezoensis* with yellow spot disease (Liu et al., 2020) and on *Delisea pulchra* with a bleaching disease (Kumar et al., 2016). The bleaching and white coloration symptom in *C. lentillifera* may not only be linked to a pathogen invasion but also an excessive oxidative burst response. According to Box et al. (2008), some *Caulerpa* species produce reactive oxygen species (ROS) against invasive organisms and if the ROS production is excessive, a situation of oxidative stress appears in the macroalga.

We hypothesize that *Phycisphaera* and *Algisphaera* spp. of the order Phycisphaerales were opportunistic pathogens or saprophytes of *C. lentillifera*. As mentioned before, the order Phycisphaerales was typical of the disease cases from the Chinese aquaculture, and strains of these genera were isolated from marine macroalgae presenting agarolitic activity (Fukunaga et al., 2009; Yoon et al., 2014). Nevertheless, Planctomycetes are widespread in the epiphytic microbial community of macroalgae, possess potential beneficial effects for their host like the production of bioactive compounds, and are rarely identified as seaweed pathogens (Lage & Bondoso, 2014). Some strains of *Phycisphaera, Algisphaera*, Pir4 lineage, and *Blastopirellula* may be using their higher number of sulfatases for the degradation of algal polysaccharides (Bondoso et al., 2017) from the dead or senescent tissue of *C. lentillifera*, which could be injured by other pathogen or degraded because of excessive production of ROS.

*Woeseia* species of the order Steroidobacterales can utilize a broad range of energy‐yielding metabolisms and substrates (Mußmann et al., 2017) and some strains may be saprophytes in *C. lentillifera*. Other bacteria may be specialized in small molecules derived from the breakdown of algal polymers and benefited from the degradation of algal tissue. Members of the family Methyloligellaceae oxidize generally one carbon atom molecules (Walker et al., 2021) and Patescibacteria (JGI 069‐P22 and some Saccharimonadales in this study) may be using monosaccharides because they have reduced genes for polysaccharide catabolism in their simple genomes (Tian et al., 2020).

The classification of potential detrimental bacteria at higher taxonomic levels in *C. lentillifera* is not clear. The order Verrucomicrobiales is well‐known as active polysaccharide degraders (Martinez‐Garcia et al., 2012) but only a few genera were linked to algal disease. For example, several OTUs of *Rubritalea* within this order were typical of *S. japonica* with symptoms of rotten hole disease (R. Zhang et al., 2020). Furthermore, the ability to metabolize algal compounds of Rhodobacteraceae in a mutualistic relationship with seaweeds may change to detrimental with some members of this family such as *Thalassobius* and *Tropicibacter. Thalassobius* spp. characterized diseased *Delisea pulchra* (Kumar et al., 2016) and were responsible for the direct lysis of red‐tide microalgae (Wang et al., 2010). *Tropicibacter multivorans* has been detected in the microbiome of *Caulerpa cylindraceae* but little information is available about its function (Rizzo et al., 2016). In this study case, *Tropicibacter* spp. might contribute to the degradation of algal polymers.

### 4.3 Potential beneficial bacteria

A beneficial role was suspected on bacteria of the order Rhodobacterales and the genus *Rhodovulum*, with the highest relative sequence abundance at each respective taxonomic level in *C. lentillifera*. Rhodobacterales are generally surface colonizers and facilitate the settlement of microbial communities with the production of extracellular polymeric substances (Dang et al., 2008). According to Simon et al. (2017), marine Rhodobacteraceae are characterized by genes for the degradation of sulfated polysaccharides from algae, production of phytohormones, metabolism of osmolytes, transport of metals, and detoxification. *Rhodovulum* species have a high metabolic versatility with the capability to degrade organic pollutants, produce polymers, and contribute with photosynthetic functions (Baker et al., 2021; Foong et al., 2019; Khandavalli et al., 2018).

*Paracoccus* is a common Rhodobacteraceae among seaweed‐associated bacteria with a key role in the cycling of nitrogen and the production of siderophores (Mei et al., 2019). *Paracoccus* strains promote the growth and the morphogenesis in *Ulva* species (Ghaderiardakani et al., 2017) and were described as auxin producers (Kurepin et al., 2014). Plant hormone production seems to be widespread in various genera of marine bacteria (Goecke et al., 2010). Furthermore, *Paracoccus* sp. presented algicidal activity against microalgae (Zhang et al., 2018) and hypothetically, some *Paracoccus* strains may prevent microalgae fouling on *C. lentillifera*. The macroalgal tissues constitute a highly competitive niche for nutrients and space.

The presumptive production of the antimicrobial compound of *C. lentillifera* may explain partially the low diversity in healthy *C. lentillifera* compared to diseased cases. The trend of higher diversity in diseased *C. lentillifera* was reported comparing *C. lentillifera* of contrasting health states in a case from the Chinese aquaculture (Liang et al., 2019). Some *Caulerpa* species contain antibacterial compounds such as alkaloids, terpenoids, phenols, and flavonoids (Goecke et al., 2010; Yap et al., 2019; Zainuddin et al., 2019). Moreover, strains of *Clostridium sensu stricto* 7 and *Cutibacterium* might reduce diversity with the production of antimicrobial metabolites in healthy *C. lentillifera*. Some green algae provide habitat for *Clostridium* species and even for potential pathogens (Chun et al., 2013).

*Clostridium* spp. can catabolize several algal polymers (Song et al., 2011), have a likely function of copper detoxification in *Codium tormentosum* (Le Pennec & Gall, 2019), and are sources of novel antimicrobial compounds (Pahalagedara et al., 2020; Schieferdecker et al., 2019). A new thiopeptide antibiotic as a microbiota modulator was detected on *Cutibacterium* sp. (Claesen et al., 2020) and some species of this genus might produce antibiotics on *Kappaphycus striatus* (Kopprio et al., 2021). A similar role on algal defense is suspected in the other Actinobacteria *Micrococcus* spp. (e.g., Hollants et al., 2013). In addition, this genus together with *Bradyrhizobium* were reported as beneficial endophytic bacteria in many terrestrial plants with agronomic value (Afzal et al., 2019).

*Bradyrhizobium* and *Allihoeflea* of the order Rhizobiales may participate in the cycling of nitrogen and the production of phytohormones. “Rhizobacteria” were extensively studied in terrestrial plants because of their ability to fix nitrogen and to modify genetically their host. Some organisms of the family Rhizobiaceae presented growth‐enhancing and probiotic properties on green microalgae (Rivas et al., 2010). Furthermore, an endosymbiotic bacterium of this taxa was responsible for the nitrogen supply in *Caulerpa taxifolia* (Chisholm et al., 1996). *Bradyrhizobium japonicum* increases the biomass and starch content of the green microalgae *Chlamydomonas reinhardtii* (Xu et al., 2016) and some strains of *B. japonicum* are producers of several phytohormones such as indole‐3‐acetic acid, gibberellic acid, zeatin, abscisic acid, and ethylene (Boiero et al., 2007).

A beneficial role for *C. lentillifera* growth and health is hypothesized on *Brevundimonas* strains. Members of this genus establish a symbiotic relationship with green microalgae promoting their growth, producing indole‐3‐acetic acid, and enhancing nutrient uptake (Sforza et al., 2018; Tate et al., 2013; Zhang et al., 2021). Some *Brevundimonas* strains have potential antifouling activity against cyanobacteria (Lin et al., 2014) and alleviate toxicity, fix nitrogen, and promote growth in some terrestrial plants (Naqqash et al., 2020; Singh et al., 2016). *Pelomonas* sq 1964 may contribute to a healthy status on *C. lentillifera*. This genus is common in the rhizosphere of some plants with a nitrogen fixation function (Terakado‐Tonooka et al., 2008) and some of their aquatic strains produce antibacterial compounds such as pelopuradazole (He et al., 2014). Surprisingly, potential human pathogens like *Escherichia‐Shigella* and *Staphylococcus* were related to the healthy state of *C. lentillifera* according to SIMPER analysis. Hollants et al. (2013) reported these bacteria with the beneficial functions of defense and morphogenesis in seaweeds, respectively.

## 5 CONCLUSIONS

A common composition of bacterial communities was observed in diseased *C. lentillifera* from Vietnamese and Chinese study cases. A disease in *C. lentillifera* may be caused by a community of detrimental bacteria together with changes in algal response, and not only by a particular pathogen (e.g., Kumar et al., 2016). Moreover, in our study was not possible to differentiate pathogens from saprophytes. A mutualistic relationship with particular bacteria may change and become detrimental under certain conditions; nevertheless, clear differences between healthy and diseased states were observed at several taxonomic levels. This study explores changes in the bacterial community composition of *C. lentillifera* under two conditions, detects co‐occurrence but not causality, we cannot discard other etiological agents not covered by the selected amplicon. Common patterns and roles were inferred in this initial study from the Vietnamese aquaculture, a valuable insight for the understanding of potential key players in the sea grape holobiont. The microbiome research on *C. lentillifera* is still in its infancy and the results of this study are valuable but limited, we recommend further basic and applied research using NGS techniques on culturable and nonculturable microorganisms. Bioactivity studies may confirm the antimicrobial properties *of C. lentillifera* and attached microorganisms, and shotgun metagenomics, metatranscriptomics, and metabolomics may provide valuable insights into the functions of the *C. lentillifera* microbiome and potential biotechnological applications.

## CONFLICT OF INTERESTS

None declared.

## ETHICS STATEMENT

None required.

## AUTHOR CONTRIBUTIONS

**Germán Kopprio**: Conceptualization (equal), data curation (equal), formal analysis (equal), investigation (equal), methodology (equal), project administration (equal), writing‐original draft (equal). **Nguyen Dinh Luyen**: Investigation (equal), methodology (equal). **Le Huu Cuong**: Conceptualization (equal), Investigation (equal), methodology (equal). **Tran Mai Duc**: Investigation (equal), methodology (equal). **Anna Fricke**: Investigation (equal), methodology (equal), visualization (equal), writing‐original draft (equal). **Andreas Kunzmann**: Conceptualization (equal), funding acquisition (equal), investigation (equal). **Le Mai Huong**: Funding acquisition (equal), investigation (equal), supervision (equal), visualization (equal). **Astrid Gärdes**: Conceptualization (equal), formal analysis (equal), funding acquisition (equal), investigation (equal), methodology (equal), project administration (equal), supervision (equal), visualization (equal).

## Data Availability

Sequencing data are available at European Nucleotide Archive (https://www.ebi.ac.uk/ena/data/view/PRJEB42826).
